# Challenges in prokaryote pangenomics

**DOI:** 10.1099/mgen.0.001021

**Published:** 2023-05-25

**Authors:** Gerry Tonkin-Hill, Jukka Corander, Julian Parkhill

**Affiliations:** ^1^​ Department of Biostatistics, University of Oslo, Blindern, Norway; ^2^​ Parasites and Microbes, Wellcome Sanger Institute, Cambridge, UK; ^3^​ Helsinki Institute for Information Technology HIIT, Department of Mathematics and Statistics, University of Helsinki, Helsinki, Finland; ^4^​ Department of Veterinary Medicine, University of Cambridge, Cambridge, UK

**Keywords:** pangenome, bacteria, gene annotation, horizontal gene transfer

## Abstract

Horizontal gene transfer (HGT) and the resulting patterns of gene gain and loss are a fundamental part of bacterial evolution. Investigating these patterns can help us to understand the role of selection in the evolution of bacterial pangenomes and how bacteria adapt to a new niche. Predicting the presence or absence of genes can be a highly error-prone process that can confound efforts to understand the dynamics of horizontal gene transfer. This review discusses both the challenges in accurately constructing a pangenome and the potential consequences errors can have on downstream analyses. We hope that by summarizing these issues researchers will be able to avoid potential pitfalls, leading to improved bacterial pangenome analyses.

## Introduction

Prokaryotic species exhibit remarkable variation in the gene content of individual genomes at both the species and lineage levels. Following early observations that a set of *Escherichia coli* genomes shared only a fraction of their genes, larger population studies led to the concept of the prokaryotic ‘pangenome’, which refers to the entire collection of genes found within a species [1, 2]. The diversity in gene content and the evolution of pangenomes is driven vertically by gene duplication and gene fusion/fission, and horizontally by the transfer of DNA through a variety of mechanisms, including direct contact between bacterial cells and the uptake of DNA from the environment. Horizontal gene transfer (HGT) is facilitated by mobile genetic elements (MGEs) such as insertion sequences (ISs), transposons, integrative conjugative elements (ICEs), integrative mobilizable elements (IMEs), plasmids and phages [3]. The dynamics of pangenome diversity plays a central role in the evolution of prokaryotes, including in niche adaptation, competition within and between species, and in the case of pathogens, the development and maintenance of antimicrobial resistance, virulence and vaccine evasion [4–6].

**Fig. 1.**
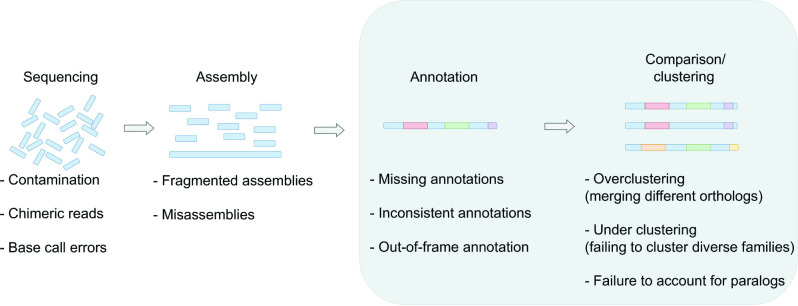
A schematic indicating the main steps in pangenome inference and the sources of error that contribute at each stage. The sections covered in this review are highlighted in grey.

Major barriers to understanding these dynamics are introduced by errors in the automated annotation, clustering and classification of orthologous and paralogous genes (Fig. 1) [7–10]. Similar to genome-wide association studies (GWASs) and phylodynamic analyses, population structure can also significantly bias efforts to understand the evolutionary dynamics of pangenomes [11–13]. This review discusses some of the major challenges these artefacts present to the analysis of bacterial pangenomes. These challenges can be broadly divided into the bioinformatics challenges of annotating, clustering and categorizing genes and the related problem of modelling the dynamics, selection and function of genes within pangenomes. The distinct but related problem of identifying fine-scale variation such as single-nucleotide polymorphisms (SNPs) is left to other publications [14, 15]. We hope that by describing these challenges and emerging strategies for dealing with them, researchers will be able to avoid some of the major pitfalls in the analysis of prokaryote pangenomes.

## Pangenome inference

The bioinformatics challenge of inferring a bacterial pangenome can be broadly split into the problems of assembly, annotation, classification and clustering of genes and intergenic sequences. Errors introduced at each stage will propagate to later steps, which can compound their impact. Small errors in individual genomes will also compound as the data set increases in size. With the increasing interest in bacterial pangenomes, methods are being developed that account for and correct these artefacts.

### Automatic gene annotation

Genome annotation is one of the central challenges in the analysis of prokaryotic pangenomes. While the scale of genome sequencing and assembly has increased dramatically over the past decade, many of the computational tools and techniques for identifying genes remain the same [8, 9, 16, 17], and are often based on the tiny, and rapidly decreasing, proportion of the data for which there is experimental evidence. Contamination, misassemblies and the difficulty of automatically annotating draft genomes lead to annotation errors. Such errors accumulate and can come to dominate gene databases, particularly if they are subsequently used to inform the annotation of new genomes [7, 8].

Prokaryote gene annotation pipelines rely on only a handful of algorithms to predict coding sequences (CDSs). These algorithms often struggle to account for fragmented assemblies, leading to misannotations and inconsistent annotations, even given identical gene sequences [7]. As most pangenome clustering algorithms only consider protein sequence, these out-of-frame errors result in erroneous orthologs. Popular algorithms such as Prodigal, Glimmer and GeneMarkS incorporate a training step that adapts the algorithm to the features of the genome being annotated [18–21]. While this improves the accuracy of prediction, it can lead to inconsistencies in the annotation of identical sequence elements when the background genetic diversity and fragmentation of each genome differs [7, 9]. Similarly, gene annotation pipelines such as Prokka, DFAST and PGAP each make use of different and in some cases user-specified reference databases, which can lead to discordance in the annotation of the resulting CDSs [22–24]. The parameter choices and post-processing steps used in each of these pipelines can also lead to annotation discrepancies [9].

Algorithms that attempt to address the issue of inconsistent annotations include Balrog and Bakta [25, 26]. Balrog is a CDS prediction algorithm, which builds a universal model of prokaryotic genes using a temporal convolutional network trained on a large and diverse set of microbial genomes. By fixing the training step, the algorithm ensures that CDSs will be called consistently in identical regions of the prokaryotic genomes [25]. The Bakta pipeline improves the consistency of annotations between runs by using a large, fixed, taxon-independent database of reference gene sequences [26]. The algorithm also includes steps to remove known spurious CDSs and small open reading frames (sORFs). However, the pipeline relies on Prodigal to call the initial CDS regions and thus previously unobserved inconsistencies introduced at this stage will persist.

Recent advances, such as the Balrog and Bakta methods explained above, are improving our ability to automatically and consistently annotate genes in draft prokaryote assemblies. The improved consistency of these approaches usually comes at the cost of fixing reference or gene prediction models trained on historical data sets: some real genes will have properties divergent from the models used in these programs, such as short genes or those with alternative codon usage biases, and will be misannotated by automated systems. Substantial challenges remain in developing methods that are both able to maintain the consistency of gene annotations and adapt to improved databases, larger numbers of genomes and the identification of previously unobserved genes, or those with anomalous properties.

### Clustering orthologs and paralogs

Following annotation, gene sequences need to be clustered into orthologous and paralogous groups. The most recent common ancestor of orthologs can be traced back to a speciation event, whereas paralogs trace their most recent common ancestry to a gene duplication event. Generating accurate clusters is critical to understanding which genomes share common genes and the evolution of pangenomes. Errors in gene annotation, contamination and the wide variation in the diversity of different gene families present considerable challenges in generating accurate clusters.

Pangenome clustering algorithms often make use of clustering or homology detection algorithms such as blast, CD-HIT and mmSeqs2 [27–29]. These are used to generate initial clusters or, in the case of blast, a pairwise distance matrix. An important, but sometimes underappreciated distinction between these tools and pangenome clustering algorithms is that they do not account for paralogous genes or the varying sequence identity of different gene families. Varying the parameters for defining orthologs versus paralogs can have significant effects on the calculation of pangenome sizes [30].

Initial efforts to address this problem primarily focused on accounting for the variance in sequence identity between different gene families [31, 32]. blast or similar fast alignment algorithms are used to generate a distance matrix between all gene pairs. Clustering is then performed using either the Markov clustering algorithm (MCL) or by looking at triangles of best hits [33, 34]. To account for the increasing size of databases, more recent algorithms precluster genes to reduce redundancy prior to generating a distance matrix [30, 35, 36]. Paralogs are usually determined using gene synteny [7, 30, 35] or by considering gene family phylogenies [36, 37]. A number of algorithms do not attempt to resolve paralogous clusters [31, 38].

The importance of accounting for annotation errors as part of the clustering process has led to the development of new algorithms. The Panaroo algorithm uses gene synteny to identify fragmented genes, missing annotations, out-of-frame errors and contamination [7]. The Peppan algorithm performs an initial clustering step before reannotating all genomes to ensure that annotations are consistent [37]. PPanGGoLiN uses gene synteny to correct for fragmented genes but does not account for other sources of error, including paralogs and variance in the sequence diversity of different gene families [38].

While these algorithms can reduce the impact of annotation errors, it is almost certain that some erroneous clusters will remain. Thus, it is essential that downstream analyses account for such errors to avoid biasing our understanding of pangenome dynamics. The largest predicted pangenome is not necessarily the most accurate.

### Intergenic regions

Prokaryotic pangenome analysis tools focus almost exclusively on protein-coding sequences. This protein-centric approach is problematic, as it neglects non-coding RNAs and many important features found in intergenic regions (IGRs), such as promoters, terminators and regulatory binding sites. These features have been shown to be under selection and can have important phenotypic implications [39, 40].

Unlike most protein identification tools, algorithms designed to annotate non-coding regions typically rely on prebuilt feature models and do not suffer from the same genome-specific training problems [41–43]. However, erroneous coding annotations can overlap with predicted non-coding RNAs, leading to similar issues [22, 26]. The impact of fragmented assemblies and contamination on the annotation of non-coding sequences is similar and is likely to lead to considerable sources of error.

The clustering of non-coding regions has received relatively little attention. In the majority of cases, intergenic features are clustered using standard clustering algorithms such as CD-HIT and mmSeq2. As stated previously, these tools do not account for errors, gene synteny or differences in the diversity of different intergenic regions and often require modifications to account for in-frame stop codons found in pseudogenes. In contrast, the Piggy algorithm uses gene synteny to classify intergenic regions and implements a similar clustering strategy to Roary. Importantly, Piggy identifies ‘switched’ intergenic regions upstream of conserved genes [44]. While the Piggy algorithm presents a major advance over classic sequence clustering algorithms, its reliance on Roary for the initial gene clustering implies that it will still be impacted by annotation errors. Improvements in the annotation, clustering and analysis of intergenic regions are essential to developing an accurate picture of the dynamics of prokaryotic pangenome evolution.

## Pangenome dynamics

Modelling the dynamics governing the evolution of bacterial pangenomes is essential to understanding how bacterial species evolve and adapt. Improved annotation algorithms and error-aware gene clustering pipelines can substantially reduce the rate at which errors introduce artificial orthologous and paralogous gene clusters or gene deletions in bacterial pangenome analyses. Nevertheless, regardless of the initial bioinformatics pipeline, it is likely that a number of erroneous gene clusters remain. This presents a considerable problem as most downstream methods for analysing bacterial pangenome dynamics do not account for errors.

The impacts of population structure and sampling bias are also often neglected in pangenome models. Similar to GWAS and phylodynamic analysis, failing to consider population structure can significantly bias results, leading to incorrect interpretations [11–13].

### Defining the core genome

Orthologous and paralogous gene clusters are classified by how common they are in a particular species. The two most common classifications are: ‘core’, which refers to genes present in all or almost all genomes of a taxonomic unit (usually a species), and ‘accessory’, which refers to genes present in only a subset of genomes. Various sub-categories are also frequently used, including ‘rare’ genes that are observed in a single genome as well as the ‘soft core’, ‘extended core’ or ‘stabilome’, which refer to genes observed in the majority of genomes [35, 38]. These categories allow for a small amount of variation caused by assembly, annotation or clustering errors.

Typically, gene clusters are classified into categories based on predefined thresholds on the fraction of genomes they appear in. The default setting in Roary, one of the most popular pangenome analysis tools, uses a threshold of 95 % to classify core genes [35]. A reliance on strict thresholds does not allow for variance in the error rate between analyses, or the fact that errors multiply with increasing size of the data set, and fails to consider information on the underlying temporal and genetic diversity of the genomes being considered. As an example, a strict threshold is likely to classify most genes in a hospital outbreak of a bacterium as core but would classify far fewer core genes when analysing a diverse set of samples from the same species representing thousands of years of evolution.

To avoid the use of arbitrary thresholds, the PPanGGoLiN pangenome pipeline uses an expectation–maximization algorithm to partition gene clusters into a number of groups based on their presence–absence patterns and gene synteny [38]. The approach can use either a predefined number of gene categories or the number can be estimated as part of the analysis. This is a major improvement over the use of arbitrary thresholds as the algorithm is able to adapt to the error rate and dynamics of different data sets. However, other than by considering gene presence and absence, the algorithm does not incorporate information on the genetic diversity of the underlying samples, which makes it challenging to compare results between data sets.

Accounting for population structure in determining the core genome is critical to understanding whether analysis results from one data set can be generalized to a wider population [45]. Similar to determining the date of the most recent common ancestor, it is not possible to generalize results beyond the set of lineages that are represented in a sample. A potential alternative strategy is to consider gene essentiality instead of gene conservation. Essential genes are necessary for the survival of a bacterium. This definition is appealing as it is less sensitive to sampling biases. However, it introduces new challenges, as determining whether a gene is essential requires expensive and time-consuming experiments and essentiality can also be growth condition- and lineage-dependent [46]. New methods and techniques are needed to more concretely define what a ‘core’ genome is that better accounts for both population structure and erroneous gene clusters.

### Gene exchange rates and open vs closed pangenomes

The rate at which genes are gained and lost forms a critical component of the dynamics of pangenome evolution and relates directly to the diversity of genes that can be found in a genome of a lineage or species [47]. HGT also has important implications for a bacterium’s ability to adapt to new niches and, in the case of pathogens, interventions such as vaccines and drug treatments.

**Fig. 2.**
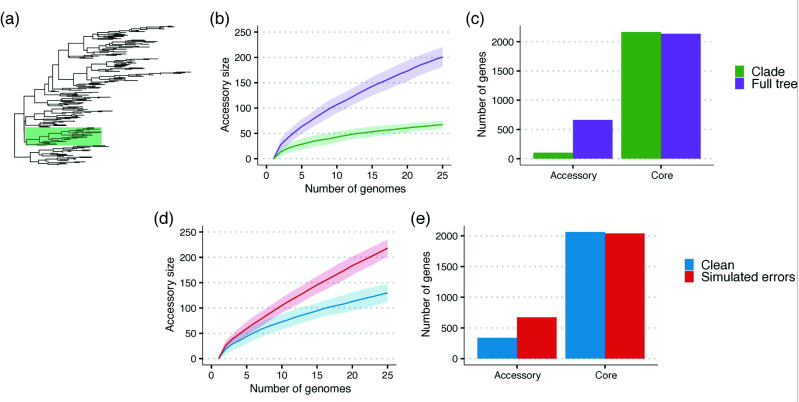
The impact of annotation errors (**d–**e) and population structure (**a–**c) on rarefaction curves and the definition of the core genome. The simulated dynamics of the red and blue data sets in (d, e) are the same. The introduction of errors, including incorrect and missing annotations (red), biases these plots. This leads to the incorrect conclusion that the parameters driving the dynamics differ between the two data sets. Similarly, the green curve and bar indicate the results if only the clade in (a) is considered versus the entire phylogeny, shown in purple. The underlying population structure leads to the inference of a larger core genome and lower gene diversity, which may cause a misinterpretation of the pangenome dynamics, despite the parameters of the two data sets being identical.

Rarefaction curves are commonly used to investigate differences in gene diversity and the rates of HGT in bacterial pangenomes. This approach is taken from ecology, where the number of unique species identified is plotted against the number of samples taken. In such studies, false positives or the misidentification of new species is rare, as such classifications are usually heavily scrutinized (as in the case of the platypus [48]). In contrast, gene annotation errors are frequent and can significantly bias these plots (Fig. 2d, e). In ecological studies, the number of samples is usually representative of the sampling effort. The same is generally not true for bacterial genomic studies, where samples are often collected from hospitals or other convenient locations leading to strong population structure (Fig. 2a–c).

In addition, to quantify the diversity of bacterial pangenomes, researchers are often interested in how well this diversity has been sampled. This has led to the classification of pangenomes as either ‘open’ or ‘closed’. Open pangenomes have a diverse accessory genome, where novel gene clusters are identified with each additional genome sequenced. Conversely, a pangenome is described as closed if it has a limited accessory genome and most genes have already been observed. Typically, the binary classification of pangenomes into these two categories relies on a limited number of samples and does not consider the population structure of the sampled genomes. Instead, methods borrowed from information theory are used, such as Heaps’ law [1, 49]. Similar to estimating the date of the most recent common ancestor (MRCA) of a species, where it is only possible to estimate the date of the MRCA of the samples, it is only possible to estimate pangenome parameters for the set of sampled genomes. Ignoring both population structure and errors in the presence and absence of genes can significantly bias our understanding of the dynamics of pangenome gene diversity, gain and loss.

An alternative strategy to the use of rarefaction curves and the binary classification of pangenomes into open and closed is to consider gene gain and loss rates. This allows for simpler comparisons between data sets and can account for both population structure and erroneous gene clusters. Two common models of gene gain and loss include the finitely and infinitely many gene models (FMG and IMG, respectively). The FMG model assumes that the same gene can be gained and lost more than once and is drawn from a finite set of genes [50]. The IMG model assumes an infinite pool of available genes and that each gene can be gained at most once [51–53]. The size of collections of gene clusters (gene families) has also been modelled using a birth–death process [54]. These models usually rely on a phylogeny built from the core genome diversity to control for the underlying population structure.

While models are available (but infrequently used) to control for population structure, the impact of errors in the analysis of gene gain and loss has received less attention. Exceptions include extensions to the birth–death model of gene families that account for errors [55, 56], in addition to techniques that model gene gain and loss rates using generalized linear regression [57].

### Pangenome selection

Our understanding of the role selection plays in shaping bacterial pangenomes is still developing. A major difference between the analysis of selection in pangenomes versus allelic variation is that pangenome selection can act at both the genome level or on individual genetic elements at the ‘genic’ level. An example of this is selfish mobile genetic elements that can have both neutral and deleterious effects on the host cell’s fitness [58].

These dynamics and the fact that multiple genes are often gained and lost at once make it challenging to extend classic neutral population genetics theory to the analysis of pangenomes. Niche adaptation occurring within bacterial species with large effective population sizes has been suggested as a plausible explanation for a large amount of within-species gene variation [59]. An alternative explanation for the correlation between within-species gene diversity and effective population size is that neutral evolution leads to populations with larger effective population sizes having a greater diversity of gene content [60]. The role of genic selection is also thought to play a role in the diversity of pangenomes [58]. The contribution of each of these mechanisms to bacterial pangenome evolution is debated [61–63]. Similar to our understanding of gene gain and loss rates, both population structure and erroneous gene clusters must be considered to accurately characterize the role of selection in pangenome evolution.

Several strategies are being used to disentangle the selective forces driving the evolution of pangenomes. One approach uses the co-occurrence of genes in different lineages to look for evidence of selection. Co-occurring genes can be associated with functional categories that enhance a bacterium’s ability to survive in a particular niche [64]. Accounting for population structure is an essential step in identifying co-evolving genes [64]. Alternative approaches have used approximate Bayesian statistical methods to fit simulation models to pangenome gene presence–absence patterns [4]. While population structure is often considered in such analyses, the impacts of erroneous gene clusters are unclear. In order to obtain an accurate understanding of bacterial pangenome dynamics, future methods will need to be robust to both population structure and errors in the annotation and clustering of pangenomes.

## Discussion

Understanding the evolutionary dynamics of prokaryotic pangenomes is key to answering several fundamental questions in microbial evolution and ecology. Pangenome dynamics also have important implications for the design of interventions targeting drug resistance, virulence and vaccine evasion of major pathogens. While there has been a dramatic increase in the number of sequenced prokaryotic genomes, there have been comparatively few advances in the development of bioinformatics and modelling methods used to analyse pangenomes.

We have outlined some major challenges in the analysis of prokaryotic pangenomes. Recent advances in the prediction of protein structure and automated annotation could lead to major improvements in our ability to accurately characterize pangenomes [65]. However, while improvements in the design of bioinformatics algorithms to annotate, cluster and classify genes will certainly improve analyses, it is essential that downstream modelling strategies are robust to population structure and errors introduced in the initial bioinformatics stage. Simulation can be used as an effective tool to test the sensitivity of mathematical models to the impacts of errors in the input data. This will be critical as we develop a better understanding of the evolution of prokaryotic pangenomes and the role of selection at both the genome and gene levels.
